# Metabolic RNA Labeling: Adding a Temporal Dimension to Transcriptomics and Epitranscriptomics Analysis

**DOI:** 10.1021/acsmeasuresciau.6c00200

**Published:** 2026-08-10

**Authors:** Di Wang, Zhenzhen Zhang, Shichao Lin, Chaoyong Yang

**Affiliations:** † MOE Key Laboratory of Spectrochemical Analysis & Instrumentation, Innovation Laboratory for Sciences and Technologies of Energy Materials of Fujian Province (IKKEM), State Key Laboratory of Vaccines for Infectious Diseases, Xiang an Biomedicine Laboratory, Key Laboratory for Chemical Biology of Fujian Province, Department of Chemical Biology, College of Chemistry and Chemical Engineering, 12466 Xiamen University, Xiamen 361005, China; ‡ School of Life Sciences, 12466 Xiamen University, Xiamen 361005, China; § Institute of Molecular Medicine, School of Medicine, Shanghai Jiao Tong University, Shanghai 200120, China

**Keywords:** metabolic RNA labeling, transcriptomics, epitranscriptomics, temporal dynamics, gene expression regulation

## Abstract

Transcriptomics and epitranscriptomics analyses have provided unprecedented insights into the transcriptome-wide RNA abundance and the post-transcriptional modifications of RNA across diverse biological processes. However, conventional transcriptomics and epitranscriptomics analyses only capture static snapshots and lack temporal resolution. To address this limitation, recent advances have integrated metabolic RNA labeling with transcriptomics and epitranscriptomics analyses, enabling time-resolved analysis of the transcription and RNA modification dynamics. This Perspective aims to highlight the recent developments and the ongoing trends in metabolic RNA labeling-based chemical tools that enable time-resolved transcriptomics and epitranscriptomics analysis. Our perspectives on current limitations and future directions are also provided, with the aim of inspiring more advanced chemical tools for accurate measurement and a deeper understanding of transcription and RNA modification dynamics in health and disease.

## Graphical abstract

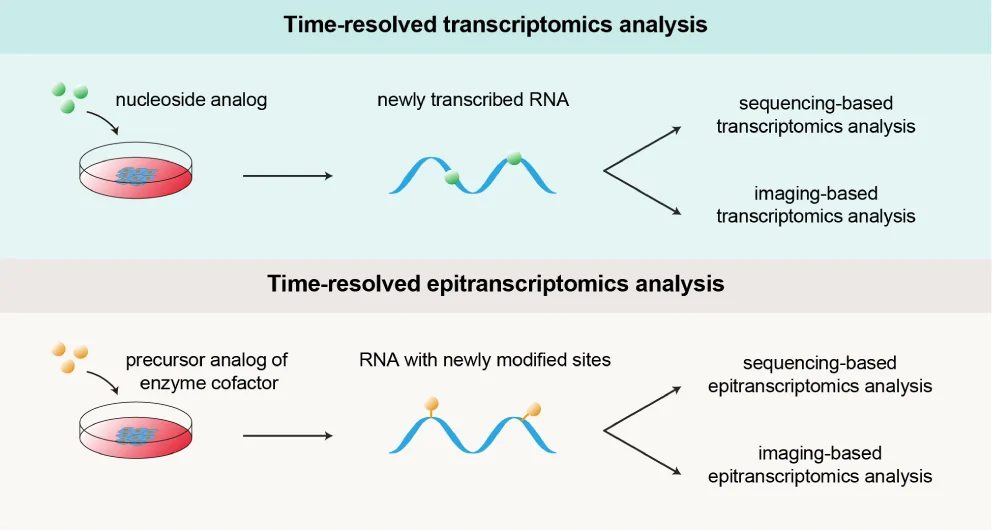

## 1 Introduction

Transcription and post-transcriptional regulation of cells are highly dynamic programs that change over time in diverse biological processes, such as cell differentiation,^1,2^ embryo development,^3,4^ disease progression,^5,6^ and response to stimuli.^7,8^ Recent advances in transcriptomics and epitranscriptomics technologies have offered unprecedented tools to profile the transcriptome-wide RNA abundance and the post-transcriptional modifications of RNA.^9–11^ However, cells can be analyzed only once due to the destructive nature of sampling in conventional transcriptomics and epitranscriptomic analysis. Moreover, the static snapshots of RNA abundance and RNA modifications at different time points convey little information about the temporal dynamics of transcription and post-transcriptional regulation. Therefore, it is necessary but challenging to develop time-resolved transcriptomics and epitranscriptomics technologies for a deeper understanding of the dynamics of transcription and post-transcriptional regulation in cells.

To address these limitations, metabolic RNA labeling has been adopted as an effective strategy to add a temporal dimension to conventional transcriptomics and epitranscriptomics analysis.^12^ Metabolic RNA labeling uses nucleoside analogues or precursor analogues of enzyme cofactors to participate in the transcription or post-transcriptional modification and introduce chemical tags in newly transcribed RNAs or newly modified sites of RNAs, respectively.^13^ These chemical tags offer unique handles for further identification of newly transcribed RNAs or newly modified sites of RNAs over a period. Compared to total RNAs and total modified sites of RNAs, newly transcribed RNAs and newly modified sites of RNAs more faithfully recapitulate the dynamics of RNA transcription and post-transcriptional modification. Therefore, metabolic RNA labeling adds a temporal dimension to transcriptomics and epitranscriptomics analyses and enables accurate measurement of RNA and RNA modification dynamics in biological processes.

In this Perspective, we first highlight typical nucleoside analogues and precursor analogues of enzyme cofactors and their principles to label newly transcribed RNAs and newly modified sites of RNAs, which lay the chemical foundation of time-resolved transcriptomics and epitranscriptomics analysis. Then, we discuss the ongoing trends of metabolic RNA-labeling-enabled time-resolved transcriptomics and epitranscriptomics analysis moving from bulk assays to single-cell assays. Finally, our perspectives on the current limitations of metabolic RNA-labeling-based time-resolved transcriptomics and epitranscriptomics analysis and the future directions for improvement are also provided. We anticipate that this Perspective will inspire chemists to develop more advanced chemical tools for accurate measurement and a deeper understanding of the RNA and RNA modification dynamics in health and disease.

## 2 The Chemistry of Metabolic RNA Labeling

Nucleoside analogues are mimics of endogenous ribonucleosides that can be taken up by cells, phosphorylated through the nucleotide salvage pathway, and incorporated into nascent RNAs during transcription.^13,14^ 5-Ethynyl uridine (5-EU) is widely used in metabolic RNA labeling-based nascent RNA analysis (Figure 1A). The bioorthogonal handle of 5-EU allows the attachment of biotin to 5-EU-labeled nascent RNAs via click chemistry and further affinity purification with streptavidin beads for sequencing. 4-Thiouridine (4sU) is another commonly used nucleoside analogue to label newly transcribed RNAs (Figure 1A). Although 4sU can form disulfide bonds with a biotin reagent for affinity purification and sequencing of newly transcribed RNAs, the most intriguing property of 4sU is its compatibility with various chemistries (e.g., alkylation,^15^ oxidative nucleophilic-aromatic substitution,^16^ OsO_4_-assisted sulfur-to-NH_2_ substitution,^17^ and addition–elimination chemistry^18^) to transform into native cytidine or cytidine analogues, yielding T-to-C substitutions of RNA reads in next-generation sequencing. Therefore, newly transcribed RNAs with 4sU-labeled sites can be directly distinguished from preexisting RNAs without physical separation.

**Figure 1:**
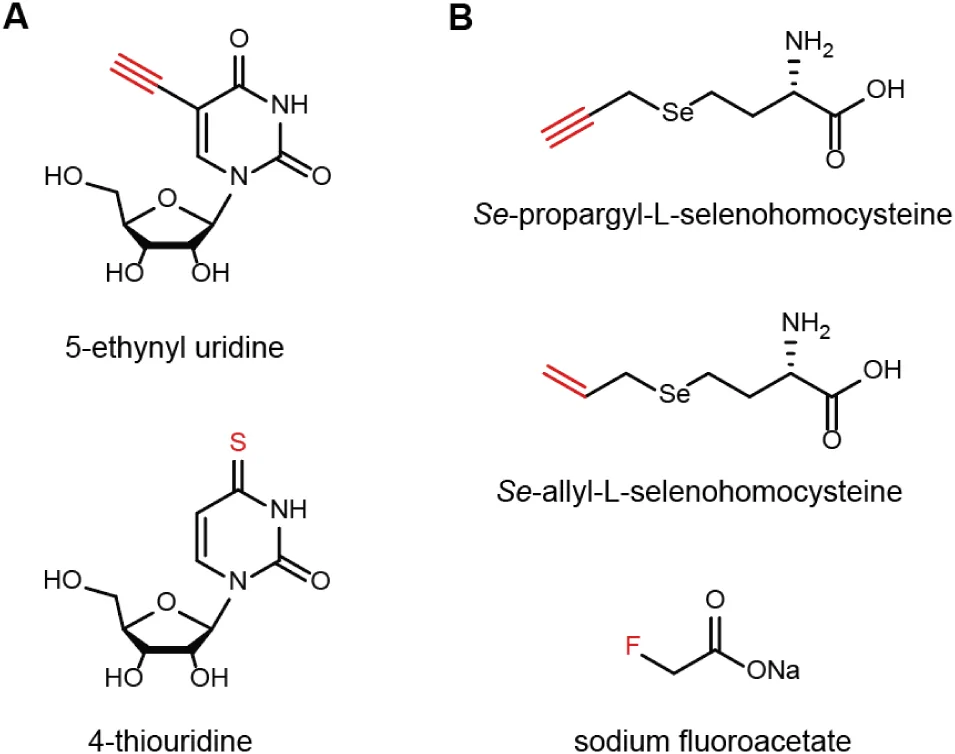
Chemical structures of typical (A) nucleoside analogues and (B) precursor analogues of enzyme cofactors used in metabolic RNA labeling.

Compared with nascent RNA labeling, metabolic labeling of newly modified sites of RNAs is more challenging. Currently, over 170 types of chemical modifications have been identified in cellular RNAs, but only a limited number of methods have been established to label newly modified sites of RNAs.^10,11^ RNA modifications rely on specific enzymes and substrates. For example, *N* ^6^-methyladenosine (m^6^A) modification, the most abundant internal mRNA modification, involves a methyltransferase writer complex and its enzyme cofactor S-adenosyl-l-methionine (SAM), transferring the methyl group from SAM to the *N* ^6^-position of a specific adenosine in RNA.^19^ Taking advantage of this property, feeding cells with methionine analogues that substitute the methyl group with an allyl or propargyl group can generate SAMs with bioorthogonal handles and thus label newly modified m^6^A sites for detection (Figure 1B).^20–23^ Similarly, newly modified *N* ^4^-acetylcytidine (ac^4^C) sites can be labeled by feeding cells with acetate derivatives that carry chemical handles (Figure 1B).^24,25^ Acetate derivatives and coenzyme A form the enzyme cofactor and acetylate target cytidine in RNAs in the presence of *N*-acetyltransferase 10 (NAT10). Therefore, newly modified ac^4^C sites are installed with chemical handles for further detection.

## 3 Metabolic RNA Labeling-Based Transcriptomics and Epitranscriptomics Analysis

### 3.1 Metabolic RNA Labeling-Based Transcriptomics Analysis

Because of the good compatibility of metabolic RNA labeling with transcriptomics analysis workflows, metabolic RNA labeling-based transcriptomics technologies have developed rapidly in recent years. For example, 4sU labeling has been integrated with massively parallel sequencing to estimate RNA transcription and degradation rates. In 4sU-seq, 4sU-labeled newly transcribed RNAs were enriched via covalent labeling and sequenced for resolving the RNA kinetics at the genome scale. TT-seq further reduced the labeling duration from 45 to 5 min, which greatly improved the temporal resolution of metabolic RNA labeling-based transcriptomics analysis. The key improvement is that TT-seq uses RNA fragmentation before the isolation of labeled RNA fragments. Thus, TT-seq could measure newly transcribed RNA fragments with a higher temporal resolution. Apart from enrichment-based methods, nucleotide-recoding methods also emerged because of the advantages of simple operation and low-input requirements. Nucleotide-recoding methods such as SLAM-seq, TimeLapse-seq, and TUC-seq exposed cells to 4sU and identified single-point mutations in RNA-seq data introduced by chemical conversion (alkylation, oxidative nucleophilic-aromatic substitution, OsO_4_-assisted sulfur-to-NH_2_ substitution) of 4sU residues into cytosine or cytosine analogues. The nucleotide-recoding chemistries of these methods are all very efficient, yielding accurate identification of newly transcribed RNAs directly. Therefore, these bulk methods lay the foundation for the further development of metabolic-RNA-labeling-based single-cell transcriptomics technologies.

As a milestone of metabolic RNA labeling-based transcriptomics analysis, time-resolved single-cell RNA sequencing (scRNA-seq) integrates metabolic RNA labeling with state-of-the-art high-throughput scRNA-seq methods.^26,27^ Metabolic RNA labeling adds a temporal dimension to scRNA-seq and enables transcriptome-wide profiling of the heterogeneous and dynamic program of gene expression. For example, 4sU-based metabolic RNA labeling has been combined with various scRNA-seq workflows (e.g., plate-based scSLAM-seq/NASC-seq,^28,29^ droplet-based scNT-seq,^30^ combinatorial indexing-based sci-fate,^31^ and microwell-based Well-TEMP-seq^32^) (Figure 2). Additional chemical conversion reaction before reverse transcription recodes base’s hydrogen-bonding pattern at the 4sU-labeled sites and introduces T-to-C substitutions after sequencing. Therefore, the number of newly transcribed RNAs with T-to-C substitutions and the number of preexisting RNAs without T-to-C substitutions can be quantified accurately for each gene in individual cells. Benefiting from the unprecedented temporal and single-cell resolution, these time-resolved scRNA-seq approaches have provided new insights into diverse biological processes, such as the onset of viral infection in host cells, the cell-state transitions in glucocorticoid receptor (GR) activation, the regulon activation in response to neuronal activity, and the early stage response to global DNA demethylation.

**Figure 2:**
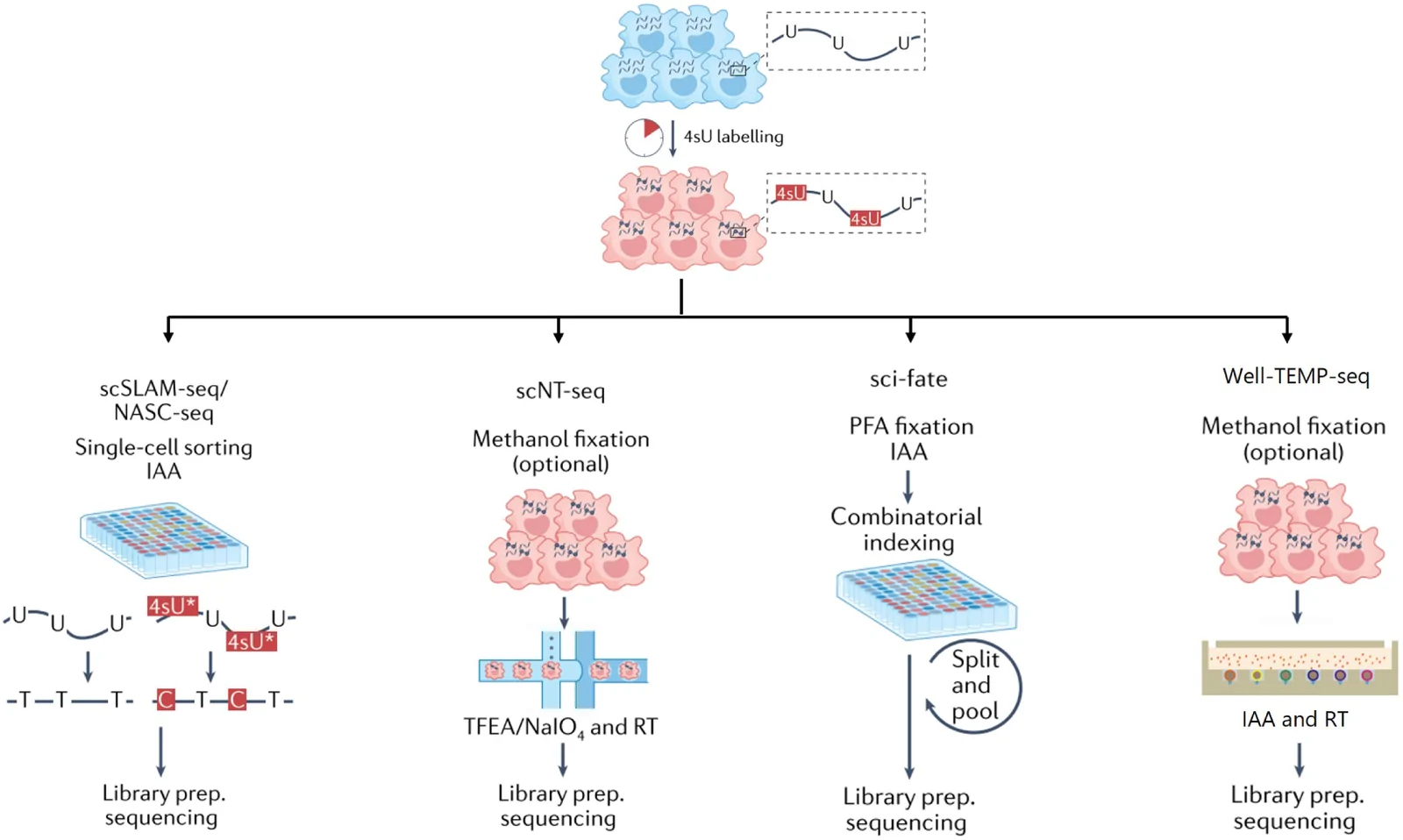
Overview of the workflows of representative metabolic RNA labeling-based time-resolved scRNA-seq methods, including plate-based scSLAM-seq/NASC-seq, droplet-based scNT-seq, combinatorial indexing-based sci-fate, and microwell-based Well-TEMP-seq. Reprinted with permission from ref 26. Copyright 2022 Springer Nature.

Although time-resolved scRNA-seq methods can provide transcriptome-wide profiling and quantitative RNA kinetic information, they lack spatial resolution. Different from time-resolved scRNA-seq, time-resolved single-cell RNA imaging, the other category of metabolic RNA labeling-based transcriptomics analysis, integrates metabolic RNA labeling with highly multiplexed imaging to profile the newly transcribed RNAs with additional spatial resolution.^33,34^ Time-resolved single-cell RNA imaging enables spatially resolved visualization of RNA dynamics at the single-cell or subcellular level. In the recently developed TEMPOmap, Ren et al. integrated 5-EU pulse-chase metabolic RNA labeling with three-dimensional in situ sequencing to simultaneously profile the spatial and temporal dimensions of individual RNA molecules (Figure 3A).^34^ Newly transcribed RNAs were first labeled by 5-EU, which added a bioorthogonal chemical handle for ligation with a splint DNA probe. After adding gene-specific padlock and primer, DNA amplicons can be generated based on the triprobe set (splint, padlock, and primer) for each targeted nascent RNA, which allows sensitive imaging of the newly transcribed RNAs with single-cell and subcellular resolution. TEMPOmap successfully resolved the subcellular RNA kinetics in various cells from transcription and translocation to degradation (Figure 3B). The full landscape of RNA subcellular kinetics at the single-cell level revealed how RNA kinetics contribute to cellular functions such as cell-cycle progression. However, TEMPOmap relies on a predefined probe set targeting known genes and suffers from limited throughput of transcriptome profiling. Besides intracellular nascent RNAs, cell surface nascent RNAs can also be profiled by metabolic RNA labeling-based time-resolved single-cell RNA imaging. Zhao’s group reported a Bioorthogonal Metabolic-to-DNA Hierarchical Converter (BMDHC) strategy that transforms cell surface nascent RNAs labeled by 5-EU into distinct fluorescence outputs (green and red) by DNA proximity hierarchical encoding. It enabled simultaneous spatial mapping of both cell surface RNA forms with the same sequence and assessment of the nascent-rate.^35^

**Figure 3:**
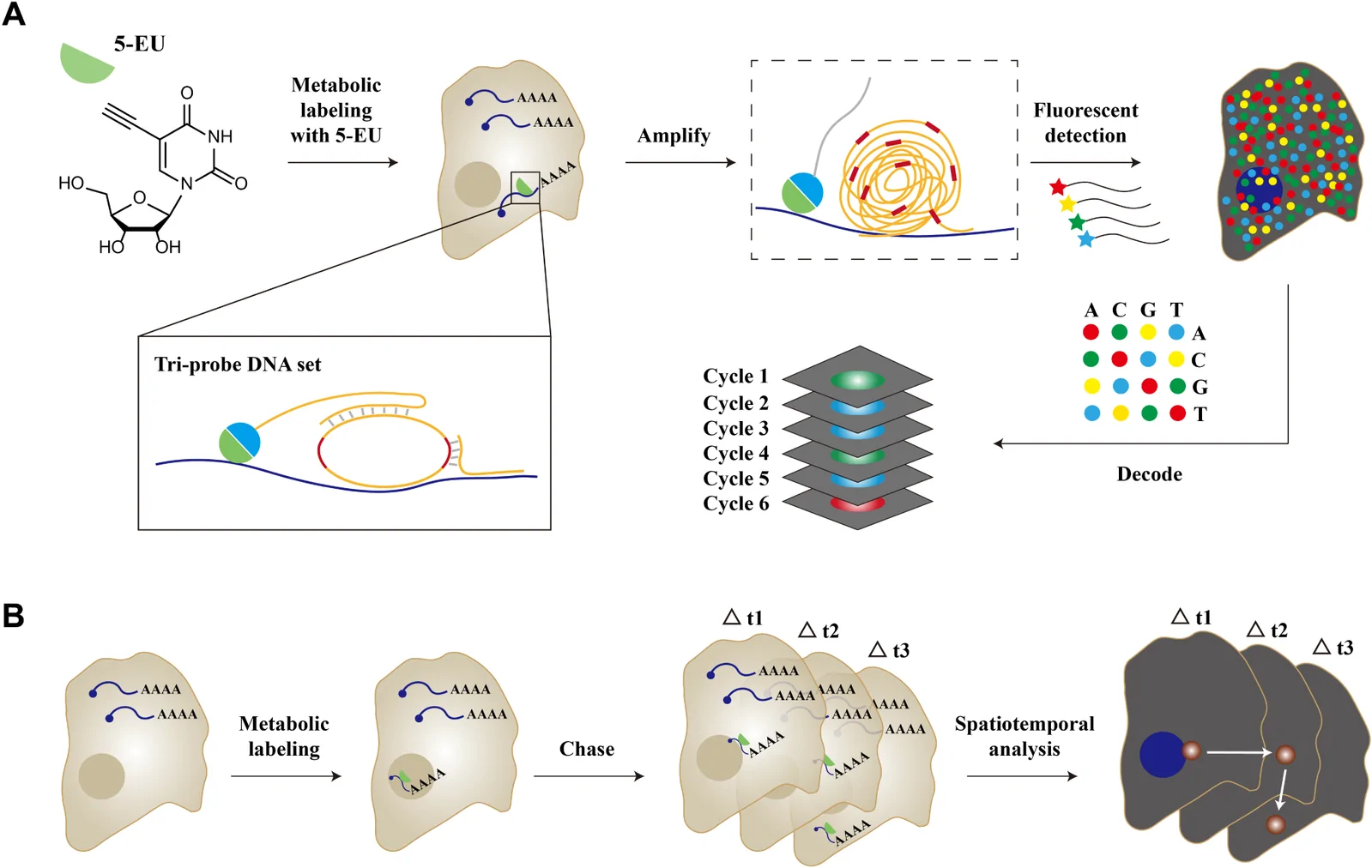
Time-resolved single-cell RNA imaging method TEMPOmap for single-cell spatiotemporal transcriptomics analysis. (A) The experimental workflow of TEMPOmap to label and detect newly transcribed RNAs with subcellular resolution. (B) The subcellular RNA kinetic landscape revealed by TEMPOmap. Reprinted with permission from ref. 33. Copyright 2024 Elsevier B.V. on behalf of KeAi Communications Co. Ltd.

In both single-nucleoside analogue-labeling-based bulk and single-cell methods, the temporal profiles of transcription and degradation rates were estimated from the abundance of both 4sU-labeled nascent RNAs and total RNAs, assuming that total RNA levels are determined by a simple (first-order) integration of production and degradation rates over time. However, they could not measure degradation in a comparably accurate way without affecting the cell viability. Further development of metabolic RNA labeling-based transcriptomics analysis extends single-nucleoside analogue labeling-based time-resolved scRNA-seq to double-nucleoside analogue labeling-based time-resolved scRNA-seq. Adopting two nucleoside analogues for labeling addresses the limitation of single-nucleoside analogue labeling-based time-resolved scRNA-seq in simultaneously capturing the RNA synthesis and degradation processes.^36^ In the recently developed scDUAL-seq, our group successfully integrated 6-thioguanosine (6sG)-4sU sequential labeling, oxidative-nucleophilic-aromatic substitution, and microwell-based scRNA-seq methods to achieve simultaneous measurement of RNA synthesis and degradation rates in thousands of single cells.^37^ The sequential labeling allows independent measurement of the RNA degradation rate and RNA synthesis rate by the time-dependent decay of 6sG-labeled transcripts and the time-dependent accumulation of 4sU-labeled transcripts in the chase periods, respectively. Moreover, inaccurate determination of RNA kinetic rates caused by the persistence of nucleoside analogues after removing the labeling medium and the internal recycling of nucleoside analogues from degraded RNAs can be avoided in double-nucleoside analogue labeling by removing transcripts with both 6sG- and 4sU-labeled sites. Therefore, double-nucleoside-analogue labeling-based time-resolved scRNA-seq has shown higher accuracy in RNA kinetic rate estimation than single-nucleoside-analog-labeling-based time-resolved scRNA-seq. The obtained RNA synthesis and degradation rates further help elucidate the fundamental biology question of how cells shape the temporal RNA dynamics by a complex and versatile interplay between RNA transcription and decay in various biological processes.

Apart from cultured cells, metabolic RNA-labeling-based transcriptomics analysis can be applied to heterogeneous cells in the tissue. Recent efforts have integrated in vivo metabolic RNA labeling (via intravenous or intraperitoneal injection of nucleoside analogues) with scRNA-seq to profile the heterogeneous and dynamic programs of cellular gene expression in tissues (e.g., kidney, brain, intestine, bone marrow, embryo, and tumor).^38–41^ The key advance is that the in vivo new RNA-based analysis allows the accurate and sensitive measurement of the temporal logic of gene regulation in complex tissues. For example, the rapid and transient transcriptional responses of the ischemia-reperfusion process in acute kidney injury, cisplatin-induced tumor-infiltrating lymphocyte responses, acute Salmonella infection-induced immune response, and early transcriptional responders in the regenerating zebrafish heart have been uncovered by the in vivo metabolic RNA-labeling-based scRNA-seq methods. Furthermore, in vivo, new RNA-based analysis adds an additional dimension to detect fine-grained cellular heterogeneity and biological function. Using the recently developed in vivo metabolic RNA labeling-based time-resolved scRNA-seq method Dyna-vivo-seq,^38^ our group uncovered RNA turnover rate (new-to-total RNA ratio) heterogeneity within proximal tubule (PT) cells of the kidney. Further analysis revealed that high-turnover subtypes of PT cells are more susceptible to ischemia-reperfusion-induced acute kidney injury. Collectively, these findings demonstrate the promise of in vivo metabolic RNA-labeling-based transcriptomics analysis in biomedical applications.

### 3.2 Metabolic RNA Labeling-Based Epitranscriptomics Analysis

Compared with transcriptomics analysis, metabolic RNA labeling-based epitranscriptomics analysis is less explored due to the complexity and diversity of epitranscriptomic modifications. Not until recently has metabolic labeling of newly modified sites of RNAs been possible for m^6^A and ac^4^C modifications.^20,24^ Hartstock et al. pioneered using *Se*-propargyl-L-selenohomocysteine (PSH), a methionine analogue with a bioorthogonal propargyl group, to label newly modified m^6^A sites in RNAs (Figure 4).^20^ As the precursor analogue of an enzyme cofactor, PSH is taken up by cells and used by methionine adenosyltransferase (MAT) to synthesize an analogue of *S*-adenosyl-l-methionine, which is further used by methyltransferase (MTase) to transfer the propargyl group to the *N* ^6^ position of adenosine. Thus, newly modified m^6^A sites can be metabolically labeled with propargyl groups. The propargyl group offers a bioorthogonal handle for conjugation with biotin-azide via click chemistry. Then, RNAs with newly modified m^6^A sites can be isolated by affinity enrichment for NGS to resolve the newly modified sites and gene information. This antibody-free approach provides a temporal dimension to epitranscriptomic analysis for capturing the dynamic m^6^A modification process. In a follow-up study, Hartstock et al. discovered that PSH could also label newly modified m^5^C sites in RNAs.^21^ Like propargyl-labeled m^6^A sites, propargyl-labeled m^5^C sites cause termination of reverse transcription during sequencing library preparation. Thus, newly modified m^5^C sites and m^6^A sites in RNAs can be simultaneously identified by examining the termination signatures after sequencing.

**Figure 4:**
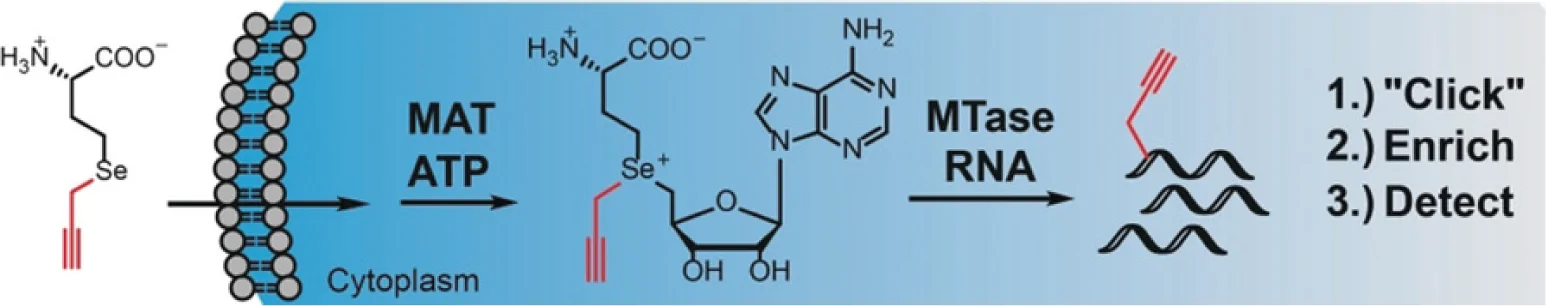
Principle of labeling newly modified m^6^A sites in RNAs using PSH for time-resolved epitranscriptomics analysis. Reprinted with permission from ref. 20. Copyright 2018 Wiley-VCH.

Similarly, newly modified m^6^A sites can be metabolically labeled with an allyl group. Liu’s group reported m^6^A-label-seq, an alternative method that feeds cells with *Se*-allyl-L-selenohomocysteine, which results in the substitution of the methyl group on newly modified m^6^A sites with the allyl group.^22^ The unique advantage of this approach is that the allyl-modified adenosine is compatible with iodination-induced cyclization, which induces misincorporation at the opposite site in complementary DNA (cDNA) during reverse transcription. Therefore, newly modified m^6^A sites can be directly identified by NGS with single-base resolution.

The strategy of using precursor analogues of enzyme cofactors to label newly modified m^6^A sites can also be applied to label newly modified ac^4^C sites in RNAs. Zhou’s group developed FAM-seq, a fluorine-assisted metabolic sequencing method to detect newly modified ac^4^C sites.^24^ FAM-seq adopts fluoroacetate, the analogue of acetate, to label cells (Figure 5A). Fluoroacetate and coenzyme A form the enzyme cofactor (fluoroacetyl-CoA) and acetylate target cytidine in RNAs in the presence of NAT10. A fluorine-thiol displacement reaction (FDTR) is further performed to label the resulting fluoroacetamides with biotin tags with a high yield for subsequent enrichment and RNA sequencing. Thanks to the ability to detect newly modified ac^4^C sites, FAM-seq provided a sensitive and accurate method to detect the differential distribution of ac^4^C sites caused by the short-term cell response to a stimulus and revealed the previously unexplored role of ac^4^C modification in regulating chemotherapy drug resistance in chronic myeloid leukemia.

**Figure 5:**
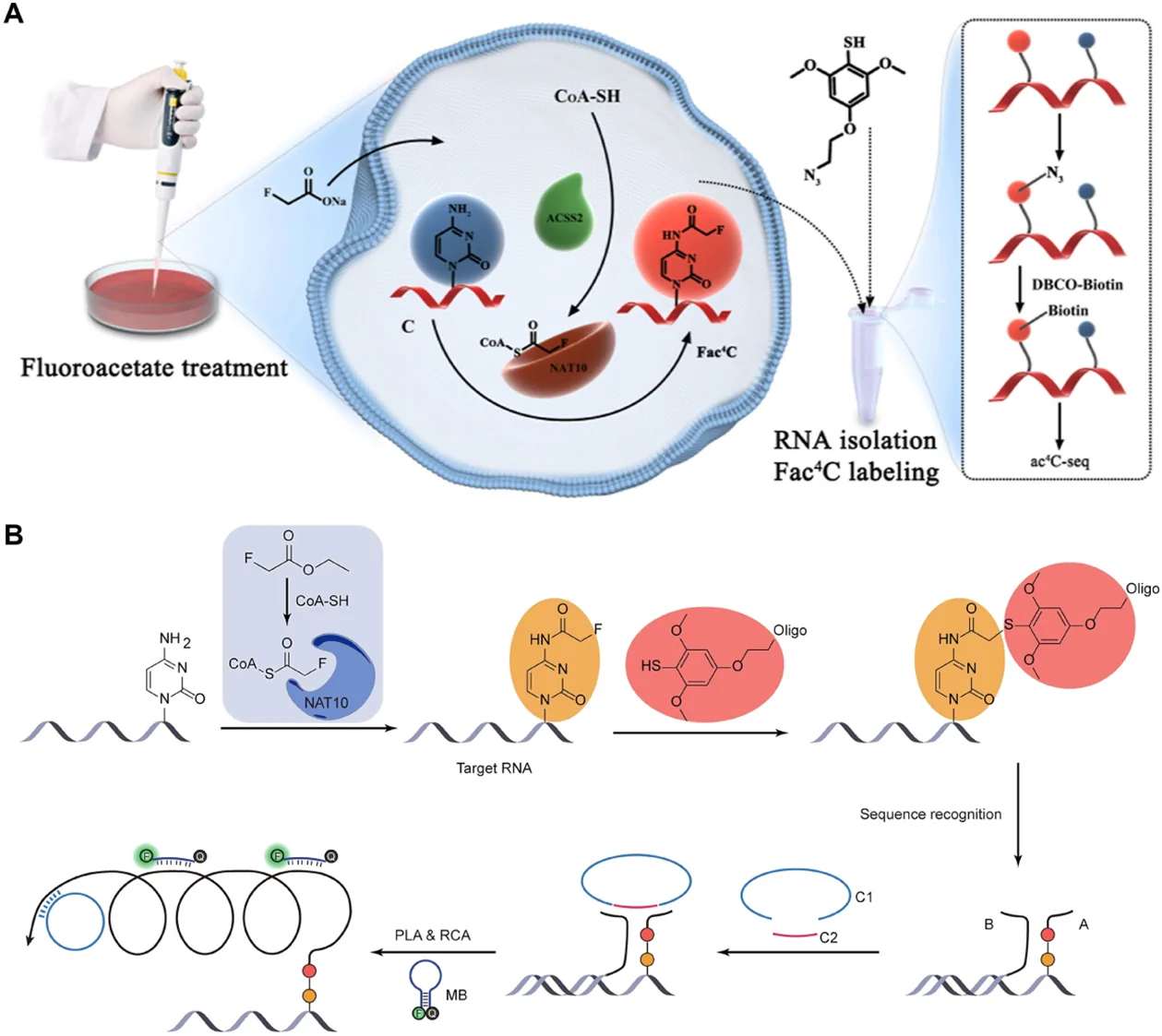
Schematic illustration of the workflows of (A) sequencing-based FAM-seq and (B) imaging-based FMPLA to label and detect newly modified ac^4^C sites in RNAs. Reprinted with permission from refs. 24 and 25. Copyright 2023 American Chemical Society and 2025 Oxford University Press.

To further resolve the spatiotemporal dynamics of ac^4^C modification in RNA, Zhou’s group developed a fluorine metabolic labeling-mediated proximity ligation assay (FMPLA), which combined the fluorine labeling with a proximity ligation-based in situ sequencing method for profiling the spatiotemporal dynamics of ac^4^C modification (Figure 5B).^25^ Newly modified ac^4^C sites labeled by fluorine offer a handle to conjugate with a DNA probe via the FDTR, which forms a circular template with a probe set that target specific region of RNA after the proximity ligation. The circular template can be amplified through rolling circle amplification (RCA) and detected by fluorescent imaging. Therefore, the abundance and the subcellular distribution of newly modified ac^4^C modifications for specific sites of specific RNAs can be measured with single-molecule resolution. In a cisplatin-treated model, FMPLA revealed significant accumulation of newly modified ac^4^C noncoding RNAs (ncRNAs) in nuclear foci, indicating a potential role of ac^4^C modification in influencing the subcellular localization, stability, and function of ncRNA during DNA repair processes.

## 4 Future Improvements and Perspectives

Although metabolic RNA-labeling-based transcriptomics and epitranscriptomics analysis have developed rapidly in the past decades, current limitations in labeling efficiency, temporal resolution, biocompatibility of labeling molecules, diversity of labeling targets, and clinical translation require future efforts for improvement.

The labeling efficiency of nucleoside analogues and precursor analogues of enzyme cofactors is critical for the accurate measurement of newly transcribed RNAs and newly modified sites in RNAs. These exogenous analogues should compete with their endogenous counterparts during the labeling processes and compete with each other in double metabolic RNA labeling, which leads to the incomplete labeling of newly transcribed RNAs and newly modified sites in RNAs. For example, the 4sU incorporation rate is about 1 in 50–200 nucleotides, resulting in the fact that up to 50% of reads originating from newly transcribed RNAs may not contain T-to-C substitutions.^28^ The potential bias of labeling may also source from nonuniform penetration in tissues, heterogeneous metabolism, and cell-type-specific uptake of nucleoside analogues. Although the new RNAs can be estimated by a binomial mixture model-based statistical correction, the accuracy of the new RNA measurement for each gene is questionable.^42^ Developing new nucleoside analogues with higher labeling efficiency and optimizing the labeling concentration and duration is urgent. For the labeling of newly modified sites in RNAs, very few precursor analogues of enzyme cofactors are available currently. An additional limitation is that the labeling efficiency of these precursor analogues of enzyme cofactors is unknown. Systematic evaluation of the labeling efficiency and a deeper understanding of the labeling kinetics are expected to help improve the labeling efficiency and thus the accuracy in time-resolved epitranscriptomics analysis.

The temporal resolution is also vital in metabolic RNA-labeling-based transcriptomics and epitranscriptomics analysis. Higher temporal resolution allows recording the rapid changes in RNA transcription and post-transcriptional modification, which are masked in conventional transcriptomics and epitranscriptomics analysis that only capture static snapshots of the steady states of RNA transcription and post-transcriptional modification. However, the increased temporal resolution and lowered signal-to-noise ratio due to a shorter labeling duration are hard to balance. Although fragmenting RNA and enriching labeled RNA by affinity purification before sequencing may partially solve this problem, the laborious protocols of biochemical separation methods require ample starting materials and could lead to the loss of the labeled RNA. Moreover, this strategy is less effective in measuring newly modified sites of RNAs because of the lower abundance of newly modified RNAs. Biochemical separation methods could not resolve newly modified sites of RNAs with single-base resolution and are prone to false site assignments. Appropriate controls, such as writer depletion, catalytic mutants, competition experiments, and orthogonal validation, are needed to justify the identification of newly modified sites in RNAs. Therefore, further improvement of the library construction protocols for metabolic RNA-labeling-based transcriptomics and epitranscriptomics analysis is needed to enhance the temporal resolution for capturing the dynamic processes of RNA transcription and post-transcriptional modification. For imaging-based transcriptomics and epitranscriptomics analysis, integrating metabolic RNA labeling with AI-assisted image analysis may help remove background noise and extract the low-abundance signals for accurate identification of nascent RNAs.

In metabolic RNA labeling, the cellular toxicity of nucleoside analogues and precursor analogues of enzyme cofactors needs to be evaluated carefully before use. These exogenous molecules may interfere with cellular metabolism by affecting diverse biological processes. Precursor analogue of enzyme cofactor, such as PSH, has been shown to label both RNA (nascent m^6^A sites and nascent m^5^C sites) and protein (nascent methylation sites) because it can serve as the substrate for multiple methyltransferases.^43^ What’s more, the pharmacokinetics and the short-term and long-term cellular toxicity of these labeling molecules in various cell types have not been well documented yet. Although short-term and low-concentration metabolic RNA labeling is better tolerated by cells, it relies on the improvement of labeling efficiency and the temporal resolution of labeling molecules.

Current metabolic labeling of newly transcribed RNAs is focused on mRNA (mRNA) and a few long noncoding RNAs with poly­(A) tails due to the ease of poly­(A)-selected library preparation. The transcriptional dynamics of noncoding RNAs without poly­(A) tails and their roles in the regulation of gene expression are less explored. On the other hand, the metabolic labeling efficiency of newly transcribed RNAs is closely related to the length of RNA as longer RNA leads to higher nucleoside analogue incorporation possibility. The short length of most noncoding RNAs increases the difficulty of labeling all the newly transcribed RNAs due to the limited incorporation efficiency of nucleoside analogues. For metabolic labeling of newly modified sites of RNAs, only a few (i.e., m^6^A, m^5^C, and ac^4^C) modifications have been proven feasible, while up to 170 types of chemical modifications in RNAs remain untouched. The major challenges are the limited numbers of labeling substrates and detection chemistry to transform the information on newly modified sites into detectable signals. To improve the diversity of labeling targets, it is necessary to synthesize more labeling substrates, such as the precursor analogues of enzyme cofactors, and develop more detection chemistries (e.g., covalent labeling and proximity labeling)^44^ to expand the toolbox to label newly modified sites in RNAs. Integrating metabolic RNA labeling with state-of-the-art DNA coding^45^ and programmable molecular imaging^46^ may further expand metabolic RNA labeling from temporal analysis to multidimensional molecular characterization.

To fulfill the great promise of metabolic RNA-labeling-based transcriptomics and epitranscriptomics analysis, the gap between methodological development and clinical translation needs to be bridged. The biggest obstacle is that the nucleoside analogues and precursor analogues of enzyme cofactors have not been approved for in vivo labeling in humans due to the potential toxicity and the unknown pharmacokinetics. To overcome the limitations of the labeling molecules in clinical applications, patient-derived organoids (PDOs) and patient-derived xenografts (PDXs) will provide alternative models to profile the transcription and RNA modification dynamics in human samples. They also allow evaluation of the stimulus response (e.g., stress and drug treatment) for clinical diagnosis, prognosis, and personalized medication, which will offer great value to the development of precision medicine and the progress of human health.

## 5 Conclusions

In conclusion, metabolic RNA labeling has added a temporal dimension to transcriptomics and epitranscriptomics analysis, leading to a deeper understanding of the dynamics of transcription and post-transcriptional regulation of cells. This Perspective provides a comprehensive overview of the current developments and the ongoing trends of metabolic RNA labeling-enabled time-resolved transcriptomics and epitranscriptomics analysis. We anticipate that the future development of metabolic RNA-labeling-based transcriptomics and epitranscriptomics analysis methods will have a greater impact on our understanding of the molecular mechanisms of gene expression and regulation in health and disease.

## Supplementary Material



## Notes

Table comparing the major methods of metabolic RNA labeling-based transcriptomics and epitranscriptomics analysis (XLSX)
